# Clinical significance of miR-19b-3p in patients with sepsis and its regulatory role in the LPS-induced inflammatory response

**DOI:** 10.1186/s40001-020-00408-3

**Published:** 2020-03-18

**Authors:** Huimin Xu, Xiuwu Liu, Huaijun Ni

**Affiliations:** 1 Department of Infectious Diseases, Linyi People’s Hospital, Linyi, 276034 Shandong China; 2 Department of Internal Medicine, Linyi People’s Hospital, Linyi, 276034 Shandong China; 3 Department of Surgery, Linyi People’s Hospital, No. 233, Fenghuang Street, Linyi, 276034 Shandong China

**Keywords:** miR-19b-3p, Sepsis, Clinical value, Inflammatory response

## Abstract

**Background:**

MicroRNAs (miRNAs) play important roles in the development and progression of sepsis. This study investigated the clinical value of miR-19b-3p in sepsis patients, and explored its role in regulating inflammatory responses in HUVECs cells.

**Methods:**

103 patients with sepsis and 98 healthy individuals were recruited. qRT-PCR was used for the measurement of miR-19b-3p level. Cell viability was evaluated using CCK-8. The protein levels of TNF-α and IL-6 were measured using ELISA. Receiver operating characteristic (ROC) curve and logistic regression analysis were constructed to evaluate the diagnostic and prognostic values of miR-19b-3p in sepsis patients.

**Results:**

MiR-19b-3p level was significantly reduced in the serum from patients with sepsis compared with healthy controls (*P* < 0.001). Sepsis patients in the survival group had significantly high miR-19b-3p levels compared with the non-survival group (*P* < 0.001). MiR-19b-3p was of a good value in predicting sepsis risk, and was an independent prognostic factor for 28-day survival in sepsis patients (OR = 3.226, 95% CI 1.076–9.670, *P* = 0.037). MiR-19b-3p level was negatively associated with serum levels of IL-6 (*r* = − 0.852, *P* < 0.001) and TNF-α (*r* = − 0.761, *P* < 0.001). Overexpression of miR-19b-3p alleviated LPS-induced inflammatory response of HUVECs, which was reflected by the decrease of the levels of IL-6 and TNF-α induced by LPS treatment (*P* < 0.001).

**Conclusion:**

MiR-19b-3p might be a potential biomarker for the early diagnosis and prognosis of sepsis patients. Overexpression of miR-19b-3p alleviated sepsis-induced inflammatory responses.

## Background

Sepsis is a condition caused by the inadequate response to the infection, leading to organ dysfunction. It is considered to be one of the most common causes of death among hospitalized patients in the intensive care unit (ICU) [1]. Sepsis is a systemic inflammatory reaction syndrome, accompanied by acute inflammatory responses, with the release of multiple inflammatory factors such as TNF-α and IL-6 [2]. Previously, several studies have discussed the occurrence of endothelial dysfunction during sepsis, which can further lead to thrombotic microangiopathy (TMA), remote organ dysfunction and death [3–5]. At present, the management of sepsis mainly focuses on containing the infection through source control and antibiotics plus organ function support [6]. The immediate diagnosis and intervention are of great significance for the favorable prognosis of sepsis patients.

MicroRNAs (miRNAs) are endogenous RNAs with about 23 nucleotides, which regulate target gene expression [7]. MiRNAs are involved in the regulation of various important biological processes and play crucial roles in the occurrence and development of diseases by regulating the stability or translation of miRNAs [8]. MicroRNAs have been reported to play a series of roles in the development and progression of sepsis, and numerous miRNAs have been determined to be abnormally expressed in sepsis samples, such as miR-21 and miR-26b [9–11]. MiR-19b-3p belongs to the miR-17/92 cluster of miRNAs, which is first originated from human B-cell lymphoma samples; this miRNA cluster has been found to have decisive biological significance in the development of cancer and many other pathological pathways [12, 13]. Accumulating evidence also suggests that miR-19b-3p plays an anti-inflammatory role in several human diseases, such as Crohn's disease and rheumatoid arthritis [14, 15]. In addition, miR-19b-3p is also reported to be involved in the regulation of different cell inflammatory response [16, 17]. However, limited studies have revealed association between sepsis and miR-19b-3p, which might be developed to be a reliable and sensitive biomarker for sepsis.

In the present study, a cohort of sepsis patients were recruited, and miR-19b-3p expression was determined to be downregulated in the serum of sepsis patients, we further explored its clinical values in sepsis patients. In addition, in vitro functional experiments were also performed to detect the role of miR-19b-3p in regulating inflammatory responses in HUVECs cells.

## Materials and methods

### Study population and sample collection

In the current study, a total of 103 patients with sepsis were enrolled, who were admitted to ICUs of Linyi People’s Hospital between August 2016 and December 2017. All patients were diagnosed according to the International Sepsis Definitions Conference diagnostic criteria for sepsis (2012) [18]. Patients who had the following conditions were excluded: in an immunocompromised state; pregnant; human immunodeficiency virus (HIV)-positive; receiving immunosuppressive, steroid, or radiation therapy. Another 98 healthy individuals were recruited as control group, who were proceeded with the routine physical examination in the same hospital. Within 24 h of admission to ICU, the blood samples were collected, and chronic health evaluation II (APACHE II) score and sequential organ failure assessment (SOFA) score of sepsis patients were evaluated and recorded. The clinical characteristics of the study population were collected, including age, gender, body mass index (BMI), serum creatinine (Scr), albumin, white blood cell (WBC), C-reactive protein (CRP), and procalcitonin (PCT). This study was approved by the Ethical Committee of Linyi People’s Hospital, and the written informed consent was collected from each participant.

### Cell culture and transfection

HUVECs were purchased from American Type Culture Collection (ATCC) (Manassas, VA). The cells were cultured in Dulbecco’s Modified Eagle’s Medium (DMEM, Gibco, Grand Island, NY) supplemented with 10% heat-inactivated fetal bovine serum (FBS, Gibco) at 37 °C in a 5% CO_2_ atmosphere. For LPS group, cells were administrated with LPS (100 ng/mL) for 24 h.

MiR-19b-3p mimic, miR-19b-3p inhibitor, or their negative controls (mimic NC and inhibitor NC) were chemically produced by GenePharma Co., Ltd. (Shanghai, China). The cell transfection was performed using Lipofectamine 2000 (Invitrogen, Carlsbad, CA, USA).

### CCK-8 assay

To evaluate the cell viability after different treatment, Cell Counting Kit-8 assay (CCK-8, Dojindo Molecular Technologies, Gaithersburg, MD) was performed. 10 μL CCK-8 solution was added to the cell culture medium. After incubation for 1 h at 37 °C with 5% CO_2_, the optical density was measured using a microplate reader (Bio-Tek Instruments, Winooski, VT, USA) at 450 nm. Cell viability was evaluated as the percentage of the control group.

### RNA extraction and quantitative real-time polymerase chain reaction (qRT-PCR)

The total RNA was extracted using Trizol Reagent (Invitrogen, Carlsbad, CA, USA) according to the manufacture’s protocol. Then, reverse transcription reactions were performed using the miScript Reverse Transcription Kit (QIAGEN, Hilden, Germany). The level of miR-19b-3p was measured by qRT-PCR through using a SYBR Green I Real-Time PCR Kit (GenePharma, Shanghai, China). U6 was used as an internal standard. The relative expression of miR-19b-3p was measured using the comparative delta CT (2^−ΔΔCt^) method with values normalized to the expression of U6.

### Measurement of cytokines

The protein levels of TNF-α and IL-6 were measured using ELISA kits according to the manufacturer’s protocols (R&D Systems China Co., Ltd.).

### Statistical analysis

SPSS version 18.0 software (SPSS Inc.) and GraphPad Prism 5.0 software (GraphPad Software, Inc.) were used for data analysis. Data were checked for normality via the Kolmogorov–Smirnov (K–S) normality test. If the data were consistent with the normal distribution, the result was presented as Mean ± SD. Median and interquartile range (IQR) were used to express the non-normal distribution data. Differences between two groups were calculated using the Mann–Whitney *U* test for non-normally distributed continuous variables, Student’s *t* test for normally distributed continuous variables, and Chi-squared test for categorical variables. Differences between multiple groups were compared using one-way analysis of variance (ANOVA) analysis. The association between miR-19b-3p expression and clinical parameters was assessed using Spearman’s correlation coefficient. Receiver operating characteristic (ROC) curve analysis and logistic regression analysis were constructed to evaluate the diagnostic and prognostic values of miR-19b-3p in sepsis patients. *P* < 0.05 was considered an indication of a statistically significant difference.

## Results

### Demographics of the study population

The clinical characteristics of the study population are summarized in Table 1. According to the K–S test results, data of WBC (*P* = 0.079), CRP (*P* = 0.065), and SOFA score (*P* = 0.200) in sepsis group were consistent with the normal distribution, and other data (all *P* < 0.05) were non-normal distributional. We found that there was no significant difference in age (*P* = 0.229), gender (*P* = 0.409), and BMI (*P* = 0.315) between healthy and sepsis groups. However, the sepsis group had higher levels of Scr, WBC, CRP, and PCT in the serum, while had lower serum albumin levels than healthy group (all *P* < 0.001), and the differences reached significant level.

**Table 1:** Comparison of the baseline data between the two groups of study objects

Parameters	Health (*n* = 98)		Sepsis (*n* = 103)		*P* value
	Mean (IQR)	K–S test (*P* value)	Mean (IQR) or ± SD	K–S test (*P* value)	
Age (years)	55 (43)	< 0.001	50 (44)	0.002	0.229
Gender (male/female)	58/40	–	55/48	–	0.409
BMI (kg/m^2^)	23.51 (21.75)	0.030	23.40 (21.88)	0.005	0.315
Scr (mg/dL)	1.03 (0.85)	0.037	1.63 (1.40)	< 0.001	< 0.001
Albumin (g/L)	35.15 (32.63)	0.026	24.25 (22.02)	0.007	< 0.001
WBC (×10^9^/L)	8.29 (6.83)	0.002	17.80 ± 5.95	0.079	< 0.001
CRP (mg/L)	6.94 (4.48)	0.025	107.98 ± 30.05	0.065	< 0.001
PCT (ng/mL)	0.06 (0.03)	< 0.001	8.89 (8.15)	0.011	< 0.001
APACHE II score	–		13.50 (11.00)	0.002	–
SOFA score	–		5.74 ± 1.41	0.200	

Mean ± SD was used to express the normal distribution data, and median and interquartile range (IQR) were used to express the non-normal distribution data

*BMI* body mass index, *Scr* serum creatinine, *WBC* white blood cell, *CRP* C-reactive protein, *PCT* procalcitonin, *APACHE* acute physiology and chronic health evaluation, *SOFA* sequential organ failure assessment

### Serum miR-19b-3p level is reduced in sepsis patients

**Fig. 1:**
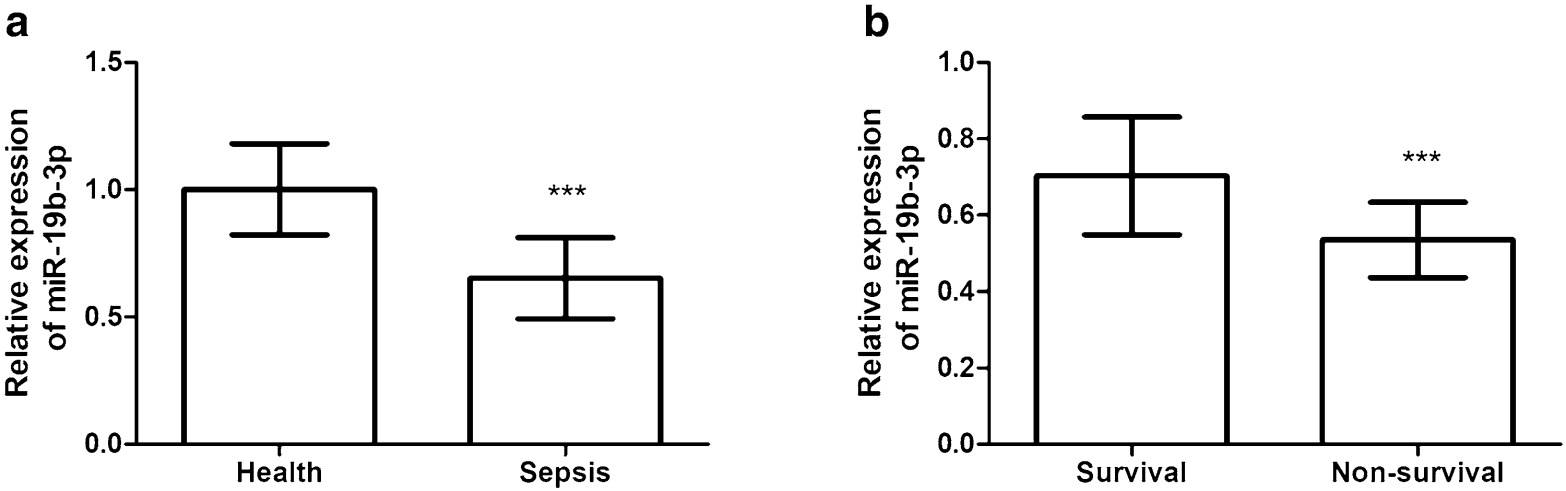
Serum expression of miR-19b-3p in sepsis patients. **a** MiR-19b-3p level was significantly reduced in the serum from patients with sepsis compared to healthy controls. **b** Sepsis patients in survival group had significantly high miR-19b-3p level compared with the non-survival group. ***P < 0.001

The serum miR-19b-3p level was compared between the healthy and patients’ groups. According to the qRT-PCR results, we observed that miR-19b-3p level was significantly reduced in the serum from patients with sepsis compared with healthy controls (Fig. 1a, *P* < 0.001). In addition, according to the 28-day survival status, all sepsis patients were further divided into survival group and non-survival group. Among 103 sepsis patients, 72 patients were survival. We further compared the miR-19b-3p levels between survival and non-survival groups. It was noted that sepsis patients in survival group had significantly high miR-19b-3p level compared with the non-survival group (Fig. 1b, *P* < 0.001). These data suggested that miR-19b-3p might have close association with the occurrence and prognosis of sepsis patients.

### Clinical value of miR-19b-3p level for sepsis patients

**Fig. 2:**
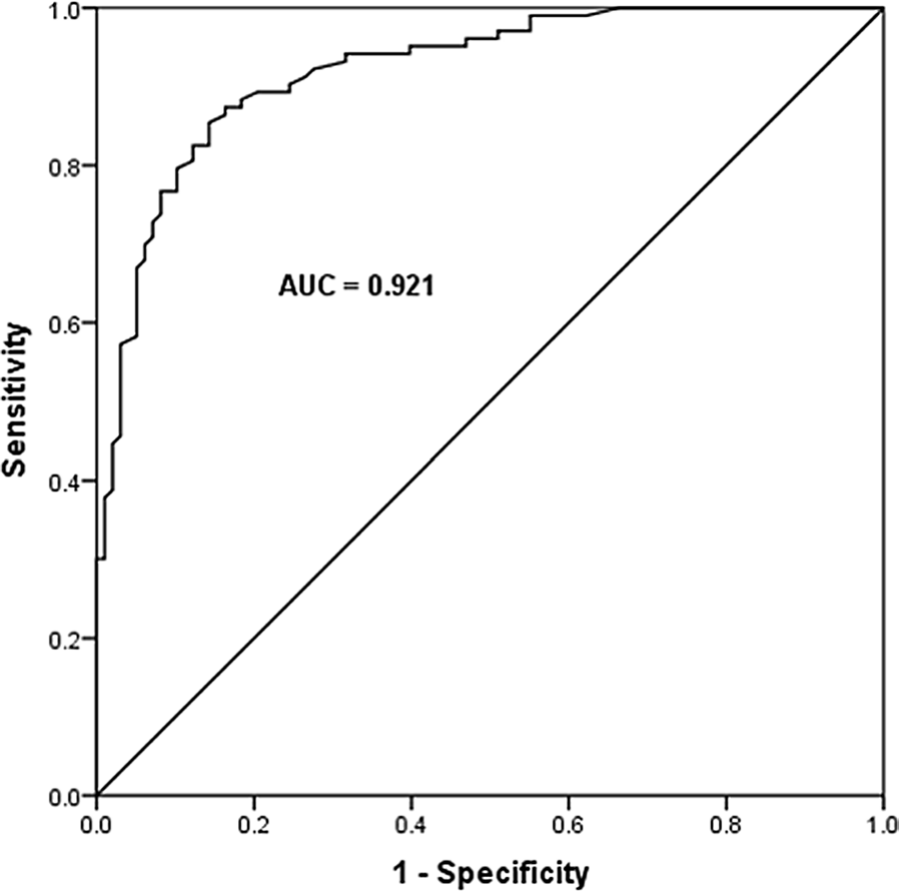
The diagnostic value of miR-19b-3p for sepsis. The AUC value was 0.921, yielding the sensitivity of 85.4% and the specificity of 85.7% at the cutoff value of 0.817

Receiver operating characteristic curve was used to determine the diagnostic value of miR-19b-3p for sepsis. As shown in Fig. 2, the AUC value was 0.921, yielding the sensitivity of 85.4% and the specificity of 85.7% at the cutoff value of 0.817.

Considering the remarkable change of miR-19b-3p expression between survival and non-survival groups, we further explored the association of miR-19b-3p with the prognosis of the sepsis patients. As shown in Table 2, the logistic regression analysis results suggested that miR-19b-3p expression (OR = 3.226, 95% CI 1.076–9.670, *P* = 0.037) and SOFA score (OR = 2.848, 95% CI 1.046–7.755, *P* = 0.041) were independent prognostic factors for 28-day survival in sepsis patients.

**Table 2:** Association of different variables with the survival of sepsis patients

Variables	OR	95% CI	*P* value
MiR-19b-3p	3.226	1.076–9.670	0.037
Age	0.426	0.150–1.206	0.108
Gender	1.280	0.432–3.792	0.656
BMI	1.454	0.494–4.278	0.497
Scr	1.475	0.537–4.055	0.451
Albumin	1.544	0.582–4.097	0.383
WBC	2.501	0.889–7.036	0.082
CRP	1.850	0.622–5.508	0.269
PCT	1.453	0.499–4.234	0.493
APACHE II score	1.533	0.521–4.511	0.438
SOFA score	2.848	1.046–7.755	0.041

*BMI* body mass index, *Scr* serum creatinine, *WBC* white blood cell, *CRP* C-reactive protein, *PCT* procalcitonin, *APACHE* acute physiology and chronic health evaluation, *SOFA* sequential organ failure assessment

### Serum miR-19b-3p level is associated with IL-6 and TNF-α levels in sepsis patients

**Fig. 3:**
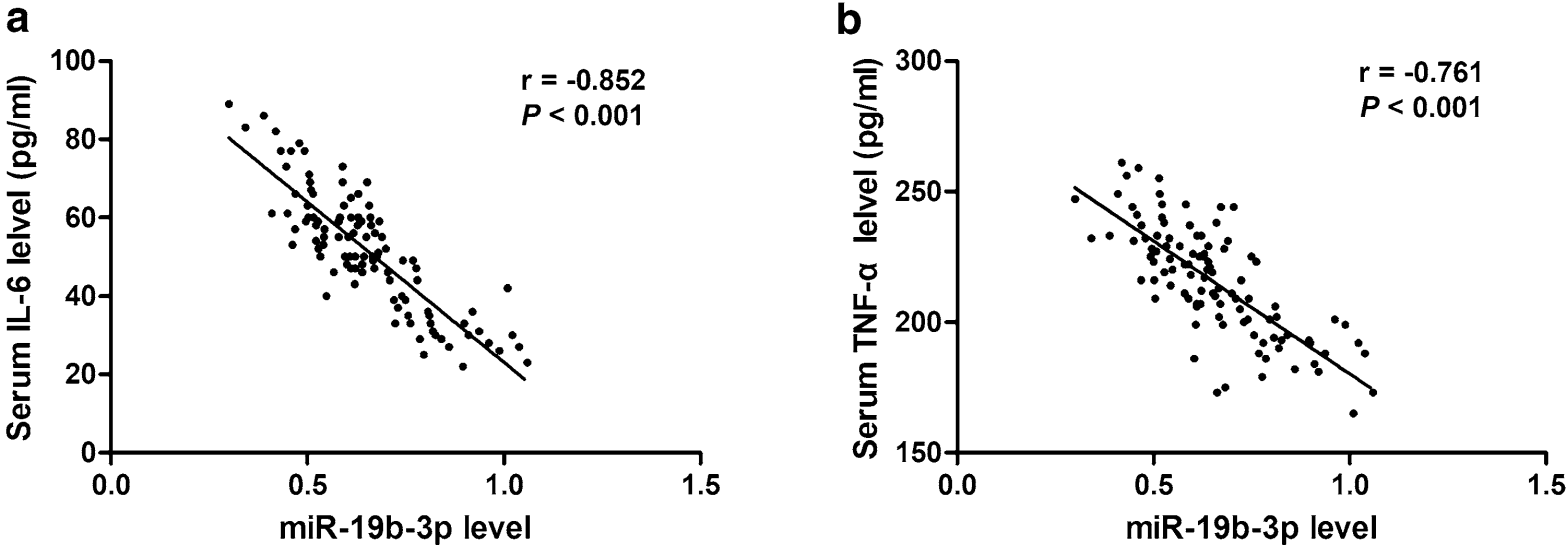
The association of serum miR-19b-3p level with the release of inflammatory factors. MiR-19b-3p level was negatively associated with serum levels of both IL-6 (**a**) and TNF-α (**b**)

Considering the crucial role of acute inflammatory responses in the development of sepsis patients, we further evaluated the association of serum miR-19b-3p level with the release of inflammatory factors, including IL-6 and TNF-α (Fig. 3). It was found that miR-19b-3p level was negatively associated with serum levels of both IL-6 (*r* = − 0.852, *P* < 0.001) and TNF-α (r = − 0.761, *P* < 0.001), revealing that miR-19b-3p might be associated with inflammatory responses for sepsis patients.

### Overexpression of miR-19b-3p alleviates LPS-induced inflammatory response of HUVECs

**Fig. 4:**
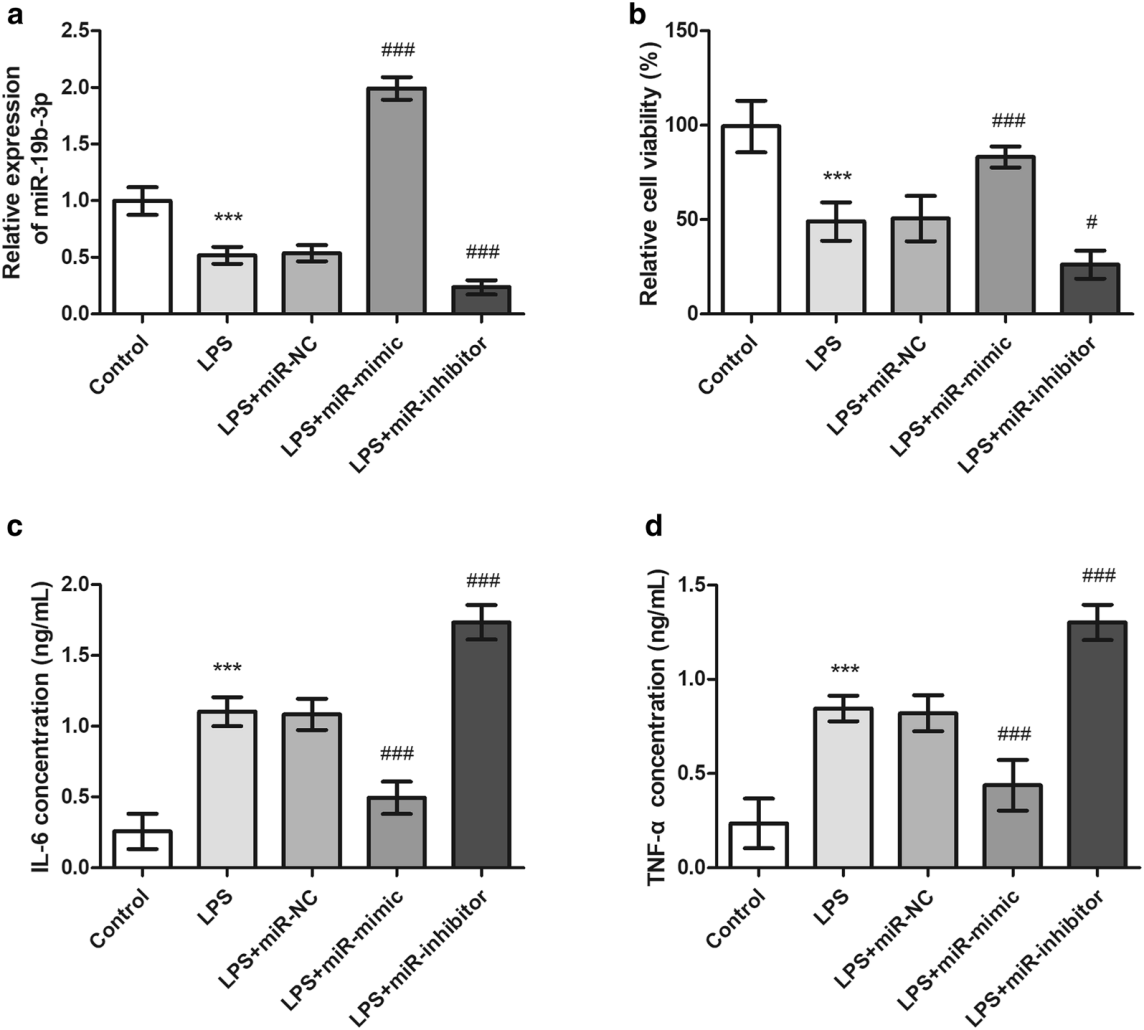
Overexpression of miR-19b-3p alleviated LPS-induced inflammatory response of HUVECs. **a** MiR-19b-3p mimic transfection significantly increased the miR-19b-3p level, whereas miR-19b-3p inhibitor transfection further aggravated the reduced level of miR-19b-3p induced by LPS. **b** Overexpression of miR-19b-3p significantly weakened LPS-induced cell viability inhibition, while miR-19b-3p downregulation aggravated the inhibitory effect of LPS on cell viability. **c**, **d** miR-19b-3p overexpression reduced the levels of IL-6 and TNF-α induced by LPS treatment, whereas miR-19b-3p downregulation intensified the inductive effect of LPS on IL-6 and TNF-α. ***P < 0.001, compared with control group; ^#^*P* < 0.05, ^###^*P* < 0.001, compared with LPS group

To investigate the role of miR-19b-3p in inflammatory responses of sepsis in vitro, miR-19b-3p levels were regulated by cell transfection in HUVECs. qRT-PCR analysis showed that LPS administration significantly reduced the miR-19b-3p level in HUVECs compared with control group. After cell transfection, it was noted that miR-19b-3p mimic transfection significantly increased the miR-19b-3p level, whereas miR-19b-3p inhibitor transfection further aggravated the reduce level of miR-19b-3p induced by LPS (Fig. 4a). In addition, CCK-8 assay was performed to detect cell viability after different treatments. As shown in Fig. 4b, overexpression of miR-19b-3p significantly weakened LPS-induced cell viability inhibition, while miR-19b-3p downregulation aggravated the inhibitory effect of LPS on cell viability. Furthermore, the ELISA results suggested that LPS treatment significantly increased the release of IL-6 and TNF-α (Fig. 4c, d). Then, the gain and lose function experiments indicated that miR-19b-3p overexpression reduced the levels of IL-6 and TNF-α induced by LPS treatment, whereas miR-19b-3p downregulation intensified the inductive effect of LPS on IL-6 and TNF-α (Fig. 4c, d). These data indicated that overexpression of miR-19b-3p alleviated LPS-induced inflammatory response of HUVECs.

## Discussion

With the improvement of the medical treatment level, sepsis is still a potentially lethal complication, and there is no special method for the treatment of sepsis. In general, laboratory hematological, biochemical, and microbiological tests are applied for the diagnosis of sepsis. But etiology diagnosis is still slow despite of new multiplex PCR assays and mass spectrometry, leading to a delay in diagnosis [19]. Furthermore, these delays contribute to a greater risk of mortality [20, 21]. Recent studies focus on the identifying of biomarkers that are helpful for the early diagnosis of sepsis, such as CRP and PCT [22]. But these tools cannot be used alone, and need the combination analysis of clinical assessment and other laboratory data. Septic shock (SS) is a serious state of sepsis patients, with relatively high mortality rate [23]. Therefore, early initiation of treatment is essential, because a delay may lead to multiple organ dysfunction [24]. It is in high demand for new biomarkers that are able to identify sepsis and SS.

Recently, the role of circulating miRNAs incited great interest to employ these miRNAs as biomarkers for various diseases. Dysfunction of miR-19b-3p regulation has been widely reported to be related to many diseases. In a study of acute myocardial infarction (AMI), circulating miR-19b-3p was consider to be a promising biomarker for the early phase of AMI [25]. Another study in Alzheimer's disease (AD) indicated that miR-19b-3p was determined to be lower in the serum of AD patients, and the serum level of miR-19b-3p might be a helpful biomarker for AD diagnosis [26]. For sepsis, a variety of abnormally expressed miRNAs have been identified to be associated with the development and progression of sepsis, such as miR-21 and miR-26b [9, 10]. In the present study, a total of 103 sepsis patients were included, and miR-19b-3p was determined to be low-expressed in the serum of the sepsis patients. Furthermore, all sepsis patients were further divided into survival group and non-survival group according to the 28-day survival status. It was noted that sepsis patients in survival group had significantly high miR-19b-3p level compared with the non-survival group. These data suggested that miR-19b-3p might have close association with the occurrence and prognosis of sepsis patients. Considering the remarkable change of miR-19b-3p expression in sepsis patients, its clinical significance in diagnosis and prognosis attracts our attention. As expect, miR-19b-3p was determined to be of a good value in predicting sepsis risk. Furthermore, logistic regression analysis results suggested that miR-19b-3p expression was an independent prognostic factor for 28-day survival in sepsis patients. However, the therapeutic data of patients was not analyzed in the current study, which may influence the results. Therefore, future studies are needed to verify the present results.

Sepsis is a lethal condition, accompanied by acute inflammatory responses, with the release of multiple inflammatory factors such as TNF-α and IL-6 [2]. Previously, numerous in vitro and in vivo studies have discussed the potential role of miRNAs in the inflammation progression [27, 28]. In the current study, miR-19b-3p level was determined to be negatively associated with serum levels of both IL-6 and TNF-α in sepsis patients. These results were consistent with the previous studies, which proved that miR-19b-3p played the anti-inflammatory role in rheumatoid arthritis and Crohn disease [14, 29]. We concluded that miR-19b-3p might be associated with inflammatory responses for sepsis patients.

Some studies have revealed that miR-19b-3p is closely related to cell inflammatory response. As Qiao et al. [16] reported, miR-19b-3p alleviated LPS-induced inflammatory injury in human intestinal cells through regulating PI3K/AKT signaling pathways. Another study in osteoarthritis reported that miR-19b-3p was involved in the regulation of LPS-induced murine chondrogenic cell inflammatory injury by inactivating Wnt/β-catenin and NF-κB pathways [30]. It is known that the endothelium plays a crucial role in health and disease, and endothelial dysfunction has been determined to contribute to sepsis pathophysiology [3]. To reverse endothelial dysfunction is considered to be an important goal for sepsis treatment. Therefore, in the present study, we investigated the role of miR-19b-3p in LPS-mediated inflammatory response in HUVECs. The gain and lose function experiments indicated that overexpression of miR-19b-3p alleviated LPS-induced inflammatory response of HUVECs. These findings suggested the involvement of miR-19b-3p in the inflammatory response for sepsis patients. However, it is unclear for the underlying mechanism of the anti-inflammatory role of miR-19b-3p in sepsis.

## Conclusion

In conclusion, the present results indicated that miR-19b-3p might be a potential biomarker for the early diagnosis and prognosis of sepsis patients. Overexpression of miR-19b-3p alleviated sepsis-induced inflammatory responses. These findings will be helpful for further understanding of the critical roles of miR-19b-3p in sepsis and may provide possible targets for sepsis diagnosis and treatment.

## Data Availability

The datasets used and/or analyzed during the current study are available from the corresponding author on reasonable request.

## Abbreviations

miRNAs: MicroRNAs
ROC: Receiver operating characteristic
ICU: Intensive care unit
TMA: Thrombotic micro angiopathy
ICUs: Intensive care units
HIV: Human immunodeficiency virus
SOFA: Sequential organ failure assessment
BMI: Body mass index
Scr: Serum creatinine
WBC: White blood cell
CRP: c-Reactive protein
PCT: Procalcitonin
ATCC: American Type Culture Collection
mimic NC: Mimic negative controls
inhibitor NC: Inhibitor negative controls
CCK-8: Cell Counting Kit-8 assay
qRT-PCR: Quantitative real-time polymerase chain reaction
ROC: Receiver operating characteristic
SS: Septic shock
AMI: Acute myocardial infarction
AD: Alzheimer's disease
